# Mortality from sepsis among middle-aged and elderly patients with pancreatic cancer: CDC WONDER 1999 To 2023

**DOI:** 10.3389/fonc.2025.1696866

**Published:** 2025-11-05

**Authors:** Aiyu Guan, Yan Chen, Yuhong Li, Weixiao Hou, Yan Liu, Tong Zhang, Haibo Yang, Peng Yao

**Affiliations:** Department of Hepatobiliary Surgery, Yuncheng Central Hospital affiliated to Shanxi Medical University, Yuncheng, China

**Keywords:** CDC WONDER, pancreatic cancer, sepsis, AAMR, AAPC

## Abstract

**Introduction:**

Sepsis-related mortality in middle-aged and older pancreatic cancer patients constitutes a significant public health issue. This study seeks to analyze trends in the age-adjusted mortality rate (AAMR) for sepsis-related fatalities among these patients in the United States from 1999 to 2023, employing data from the most recent CDC WONDER database. The temporal patterns revealed from this analysis are anticipated to guide subsequent research and public health initiatives.

**Methods:**

The CDC WONDER database was used to look at how many middle-aged and older pancreatic cancer patients in the U.S. died from sepsis between 1999 and 2023. The study utilized AAMR to evaluate temporal mortality patterns among adults aged 45 and older, categorized by race, census region, urban/rural residency, and state, using the Joinpoint regression tool. We calculated the annual percent change (APC) and the average annual percent change (AAPC), and we supplied 95% confidence intervals.

**Results:**

During the study period, the sepsis-related death rate among middle-aged and elderly pancreatic cancer patients exhibited a notable increase, with an AAPC of 2.89. Male patients consistently demonstrated a greater AAMR compared to females, with a notable increase recorded [AAPC = 2.73 (95% CI 1.61 to 3.87)]. Black or African American patients had the greatest AAMR, which also went up a lot [AAPC = 2.62 (95% CI 1.76 to 3.48)]. The mortality burden increased significantly with age, reaching its highest point in the 75–84 age range. A regional study found that the Midwest had the highest rise in AAMR [AAPC = 3.74 (95% CI 2.50 to 5.00)]. Urban people consistently exhibited a higher AAMR compared to rural communities, despite the most significant increase in AAMR occurring among rural populations [AAPC = 3.51 (95% CI 2.09 to 4.94)].

**Conclusion:**

This study’s findings reveal substantial inequalities among gender, ethnicity, age, and geographic regions. These differences show how important it is to quickly implement targeted measures to lower mortality, especially among individuals at high risk.

## Introduction

Pancreatic cancer (PC) is a highly aggressive tumor of the digestive system with a dismal prognosis, characterized by limited therapy options and frequent severe consequences, predominantly affecting middle-aged and older patients (1–3). Sepsis is a major complication that affects both the quality of life and the prognosis of patients since it happens so often and kills so many people (4, 5). The definition of sepsis, which is infection-induced, life-threatening organ dysfunction, offers a common framework for population-based surveillance and mechanistic discussions (6).Patients with pancreatic cancer are especially susceptible to secondary infections and the onset of sepsis, aggravated by tumor-induced bile duct obstruction, immunological dysfunction, and different interventions including surgery, interventional therapies, and systemic chemotherapy (7, 8). For example, those with advanced pancreatic ductal adenocarcinoma (PDAC) who also have acute cholangitis (AC) only live for an average of 4.1 months, which shows how devastating sepsis can be (4). A national study on cancer patients with infectious shock found that, even though short-term mortality rates have improved, the one-year mortality rate for pancreatic cancer patients is still quite high at 81.3% (9). Additionally, postoperative complications—particularly those of an infectious nature—significantly restrict the odds for patients to get later adjuvant treatment, further decreasing their long-term results (10).

The interaction between pancreatic cancer and sepsis establishes a complex pathophysiological network, wherein the relationship transcends mere causality, perpetuating a detrimental loop of mutual reinforcement. This network is propelled by systemic inflammation instigated by the tumor, coagulopathy, the activation of prevalent molecular signaling pathways, and the potential direct influence of infectious agents on tumor advancement (11–14).Tumor-related biological vulnerabilities may increase the risk of infection, as experimental studies have demonstrated that tumor-bearing patients are more likely to die and have higher microbial burdens and immune imbalances under comparable septic stimulation (15).

According to data from inpatient databases, sepsis in patients with pancreatic cancer is linked to a considerable increase in in-hospital mortality, complications, and resource usage (16).Even while we know more about how these things work together, there isn’t enough information in the existing literature about how sepsis affects middle-aged and older pancreatic cancer patients over time in different demographic groups. The lack of this kind of information makes it hard to come up with specific public health measures and healthcare policies that could help ease the burden of the condition. Longitudinal studies are critically required to investigate the patterns in sepsis-related mortality in pancreatic cancer patients, as this data may yield significant insights for enhancing medical practices and informing future legislation.

Our research aims to fill this void by analyzing the long-term trends of sepsis-related mortality among middle-aged and elderly pancreatic cancer patients in the United States from 1999 to 2023. We want to find patterns and risk variables that affect death rates by breaking the data down by age, race, gender, census population, and whether the person lives in a city or a rural area. There are a lot of reasons why it’s important to understand these trends. First, it will assist find groups of people who are at high risk and might benefit from focused interventions. Second, it will help us understand how well past public health interventions worked and help us plan for the future. Lastly, this study is very important for improving clinical care, guiding prevention initiatives, and finding high-risk subgroups since it shows how sociodemographic differences affect pancreatic cancer patients’ sepsis-related deaths.

## Methods

### Study setting and population

The research complies with the STROBE (Strengthening the Reporting of Observational Studies in Epidemiology) guidelines, focusing on examining mortality data related to sepsis in pancreatic cancer patients across the United States between 1999 and 2023 (17). The information came from the Centers for Disease Control and Prevention’s (CDC) Epidemiological Research Online Database (CDC WONDER), which has death certificate records from all 50 states and the District of Columbia (18). The International Classification of Diseases, 10th Revision (ICD-10) codes were used to sort death data. Codes A40 (streptococcal sepsis) and A41 (other sepsis) were used to define sepsis. ICD-10 code C25 was used to find pancreatic cancer (19). Multiple causes of death were analyzed using public-use records to examine sepsis-related mortality in pancreatic cancer patients. In patients with pancreatic cancer, listed sepsis may not always be the leading cause of death because CDC WONDER depends on death certificates. This study did not require Institutional Review Board (IRB) approval, as it utilized a publicly available, de-identified dataset provided by the government, and did not involve direct interaction with human subjects.

### Data abstraction

Information regarding the entire demographic of middle-aged and older people was gathered and classified according to gender, race/ethnicity, age brackets (45–54 years to 85+ years), census area, and urbanization stage (metropolitan vs. regions outside metropolitan areas, Health and Human Services (HHS), and the year. The 2013 National Center for Health Statistics (NCHS) urban-rural classification system was used to determine levels of urbanization (20). Counties boasting a population exceeding 50,000 were categorized as metropolitan, whereas the rest were deemed non-metropolitan. The classification of races and ethnicities encompassed Hispanic/Latino and non-Hispanic categories, which were further broken down into White, Black/African American, and various other races categorized as “non-Hispanic other.” As per the United States According to the Census Bureau, census areas were segmented into Northeast, Midwest, South, and West (21).

### Statistical analysis

CDC WONDER provided the yearly death statistics and demographic projections. The AAMR values were determined through a direct approach and aligned with the standard population of the U.S. in 2000 (22). The AAMR figures were presented for every 100,000 people, with 95% confidence intervals (CI) calculated under the assumption of a Poisson distribution. Nonetheless, in age-group stratification, the analysis was limited to basic mortality rates due to the CDC WONDER platform’s lack of support for computing age-adjusted rates for age-specific subgroups. The probable cause of this constraint is the mismatch in adapting certain age categories to fit the typical population. Despite this, basic rates continue to be effective in depicting fundamental mortality patterns. To avoid unstable estimates, we eliminated annual data units with fewer than 10 deaths; however, this suppression made it difficult to analyze longitudinal trends. As a result, we only examined the percentage change in mortality for HHS regions from 1999 to 2023. Utilizing the Joinpoint regression software (version 5.4.0, sourced from the National Cancer Institute, Bethesda, Maryland), we examined temporal mortality patterns and pinpointed meaningful statistical variations by applying the most suitable linear model to the data (23). This software employs a logarithmic-linear approach to identify major shifts in patterns over time, along with a Monte Carlo permutation technique to autonomously determine the quantity and positioning of joinpoints, thereby enhancing the model’s accuracy. In its final form, the model presents the APC for every segment at the joinpoints, accompanied by the corresponding 95% confidence interval. To determine the AAPC and its 95% confidence interval, the Joinpoint regression algorithm’s weighted mean of the APC values was employed. The AAPC encapsulates the pattern of mortality throughout the entire duration of the study. A two-tailed t-test was employed to determine the statistical importance of both APC and AAPC, maintaining a significance threshold of p < 0.05 (24).

## Results

### Overall

**Figure 1:**
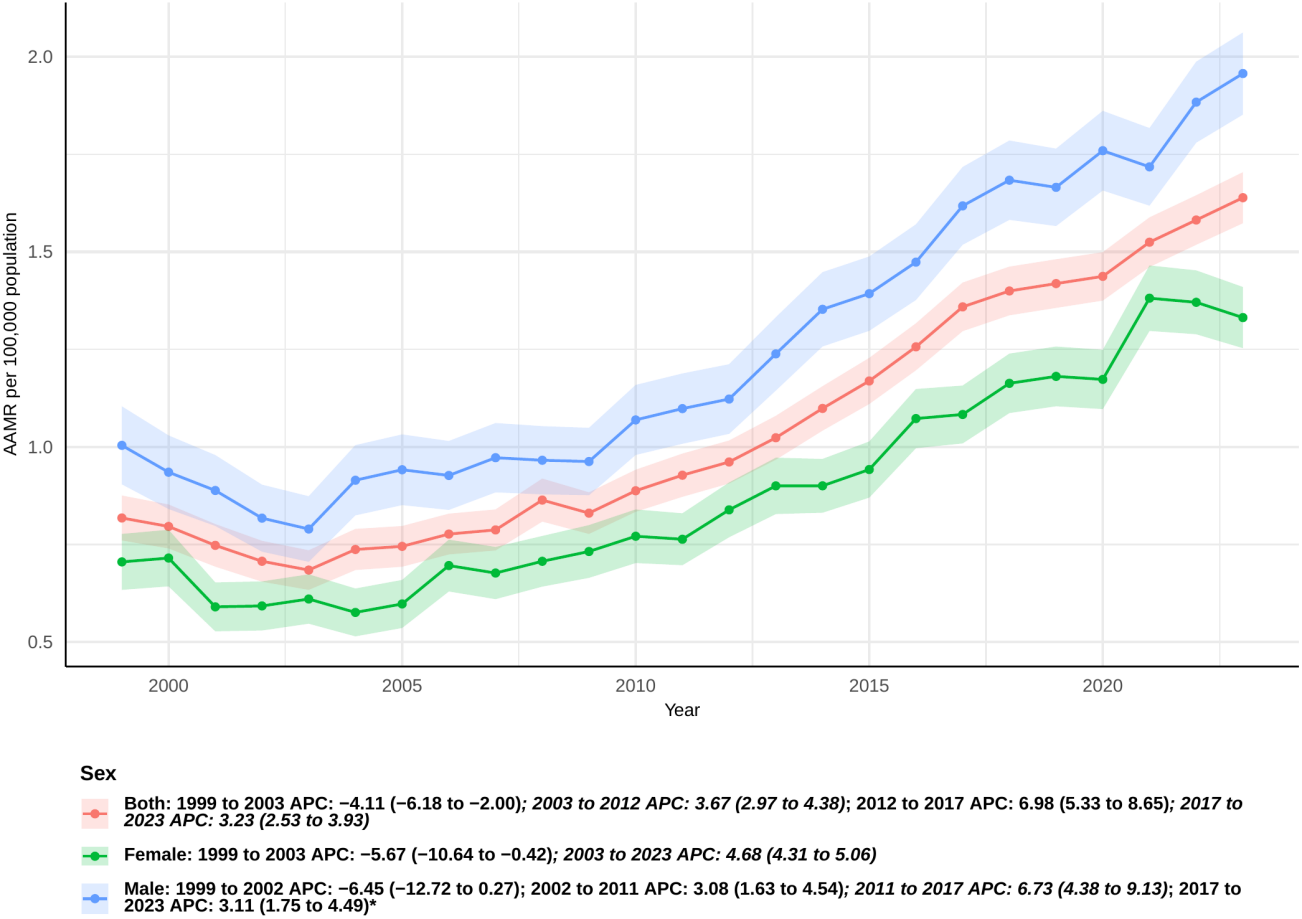
Joinpoint model of sepsis among middle-aged and elderly patients with pancreatic cancer AAMR per 100,000 residents by sex, 1999-2023. Temporal trend segments are represented by APC values. (*APC Significant. APC, annual percentage change; AAMR, age-adjusted mortality rate).

In the United States, there were 33,724 fatalities from sepsis in pancreatic cancer patients between 1999 and 2023 (**Supplementary Table 1**). The AAMR for these patients dropped a lot between 1999 and 2003 [APC: -4.11 (95% CI −6.18 to −2.00)], but then went up a lot again between 2003 and 2012, 2012 and 2017, and 2017 and 2023 [APC: 3.67 (95% CI 2.97 to 4.38), 6.98 (95% CI 5.33 to 8.65), and 3.23 (95% CI 2.53 to 3.93), respectively] (**Figure 1**). The AAPC was substantially 2.89 (95% CI 2.35 to 3.44) across the whole study period, which means that age-adjusted death rates were consistently going up (**Supplementary Table 2**).

### Sex

From 1999 to 2023, the mortality rate for sepsis was consistently greater in male pancreatic cancer patients than in female patients. From 1999 to 2003, there was a considerable drop in the number of females (APC: -5.67 [95% CI: −10.64 to −0.42]). There was a big rising tendency from 2003 to 2023, nevertheless (APC: 4.68 [95% CI: 4.31 to 5.06]) (**Figure 1**). The total AAPC for women was 2.88 (95% CI: 1.97 to 3.80) (**Supplementary Table 2**).

For males, the AAMR exhibited a non-significant decline from 1999 to 2002 (APC: -6.45 [95% CI: −12.72 to 0.27]). There were big jumps in the years 2002–2011, 2011–2017, and 2017–2023, with APCs of 3.08 (95% CI 1.63 to 4.54), 6.73 (95% CI 4.38 to 9.13), and 3.11 (95% CI 1.75 to 4.49), respectively (**Figure 1**). The overall AAPC for men was 2.73, with a 95% confidence interval of 1.61 to 3.87 (**Supplementary Table 2**).

### Race

**Figure 2:**
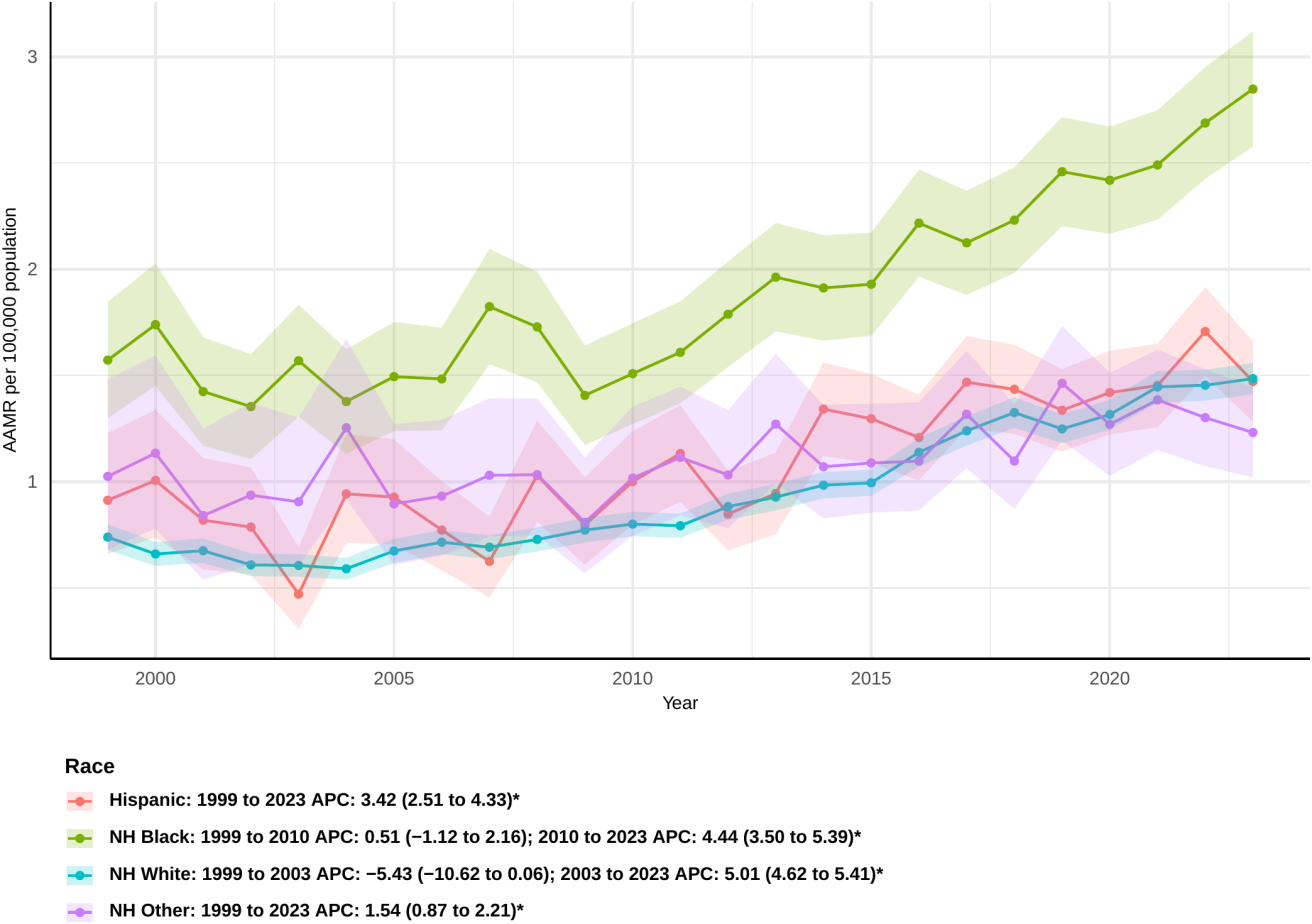
Joinpoint model of sepsis among middle-aged and elderly patients with pancreatic cancer AAMR per 100,000 residents by race, 1999-2023. Temporal trend segments are represented by APC values. (*APC Significant. APC, annual percentage change; AAMR, age-adjusted mortality rate).

When the data were analyzed by race, non-Hispanic Black or African American participants demonstrated a markedly elevated AAMR during the study period, yielding an overall AAPC of 2.62 (95% CI: 1.76 to 3.48) (**Supplementary Table 2**). The rate did go up from 1999 to 2010, but this shift was not statistically significant (APC: 0.51 [95% CI: −1.12 to 2.16]) (**Figure 2**). But from 2010 to 2023, there was a clear rising tendency (APC: 4.44 [95% CI: 3.50 to 5.39]).Hispanic or Latino persons exhibited a steady rise in sepsis-related mortality, with the most significant increase noted among all racial categories (AAPC: 3.42 [95% CI: 2.51 to 4.33]). For non-Hispanic other people, the increase was the smallest, with an overall AAPC of 1.54 (95% CI: 0.87 to 2.21). From 1999 to 2003, the death rate for non-Hispanic White people went down, although this was not statistically significant (APC: -5.43 [95% CI: −10.62 to 0.06]). After that, it went up significantly until 2023 (APC: 5.01 [95% CI: 4.62 to 5.41]). Overall, the AAPC for this group was 3.19 (95% CI: 2.24 to 4.16), indicating a consistent upward trend in mortality.

### Census regions

**Figure 3:**
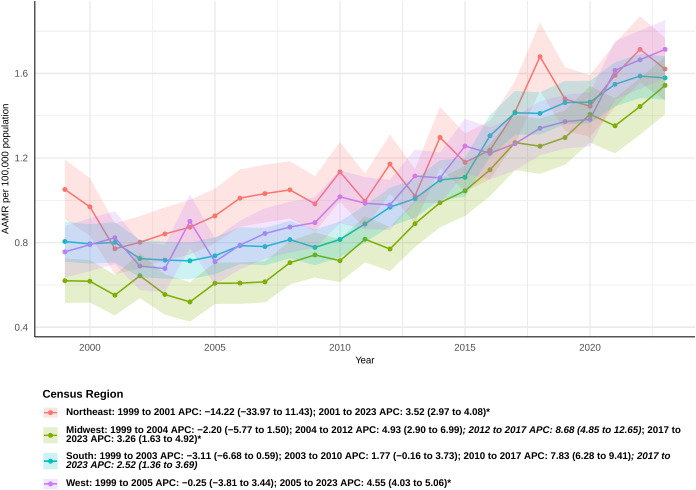
Joinpoint model of sepsis among middle-aged and elderly patients with pancreatic cancer AAMR per 100,000 residents by census regions, 1999-2023.Temporal trend segments are represented by APC values. (*APC Significant. APC, annual percentage change; AAMR, age-adjusted mortality rate).

Mortality rates linked to sepsis in pancreatic cancer patients differed among various census areas. Initially, in the Midwest, there was an inconsequential decline in the rate between 1999 and 2004 (APC: -2.20 [95% CI: −5.77 to 1.50]), succeeded by notable rises in the intervals of 2004-2012, 2012-2017, and 2017-2023. Specifically, the APCs were 4.93 (95% CI 2.90 to 6.99), 8.68 (95% CI 4.85 to 12.65), and 3.26 (95% CI 1.63 to 4.92), respectively (**Figure 3**). The overall AAPC in the Midwest was 3.74 (95% CI 2.50 to 5.00), the highest among all regions (**Supplementary Table 2**). In the South, sepsis-related mortality rates slightly declined from 1999 to 2003 and then showed a modest increase until 2010, though these changes were not statistically significant. However, significant increases were observed from 2010 to 2017 and from 2017 to 2023, with APCs of 7.83 (95% CI 6.28 to 9.41) and 2.52 (95% CI 1.36 to 3.69), respectively. The overall AAPC for the South was 2.85 (95% CI: 1.93 to 3.77).In the West, the mortality rate declined from 1999 to 2005, but this decrease was not statistically significant. A significant increase was observed thereafter, continuing through 2023 (APC: 4.55 [95% CI: 4.03 to 5.06]). The overall AAPC in the West was 3.33 (95% CI: 2.38 to 4.28). Interestingly, the Northeast was the only region that did not show a statistically significant overall AAPC during the study period. However, between 2001 and 2023, a significant increase in sepsis-related mortality was observed in this region (APC: 3.52 [95% CI: 2.97 to 4.08]).

### Urban/rural

**Figure 4:**
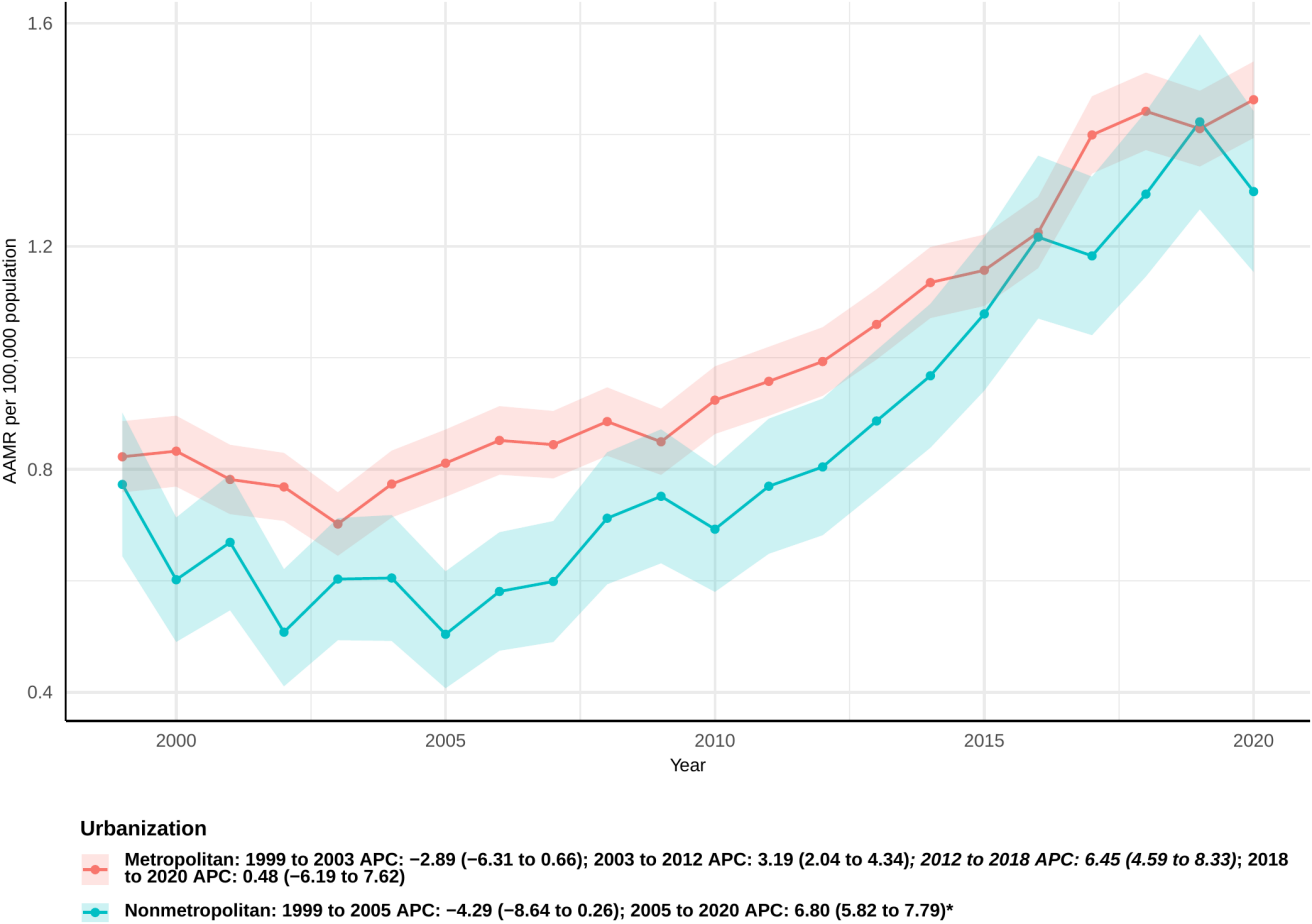
Joinpoint model of sepsis among middle-aged and elderly patients with pancreatic cancer AAMR per 100,000 residents by urban or Rural, 1999-2023. Temporal trend segments are represented by APC values. (*APC Significant. APC, annual percentage change; AAMR, age-adjusted mortality rate).

During the study period, the AAMR for urban populations remained consistently the highest, while rural populations experienced the most significant increase in the AAPC. Among urban populations, a downward trend was observed from 1999 to 2003, followed by an upward trend from 2018 to 2020; however, neither trend was statistically significant (APC: −2.89 [95% CI −6.31 to 0.66] and 0.48 [95% CI −6.19 to 7.62], respectively). Notably, significant increases were observed during the periods from 2003 to 2012 and from 2012 to 2018, with APCs of 3.19 (95% CI 2.04 to 4.34) and 6.45 (95% CI 4.59 to 8.33), respectively (**Figure 4**). The overall AAPC for urban populations was 2.65 (95% CI: 1.59 to 3.73) (**Supplementary Table 2**).

In contrast, rural populations showed a non-significant downward trend from 1999 to 2005 (APC: −4.29 [95% CI: −8.64 to 0.26]), followed by a significant increase through 2020 (APC: 6.80 [95% CI: 5.82 to 7.79]). The overall AAPC for rural populations was 3.51 (95% CI: 2.09 to 4.94), representing the largest increase among all population groups.

### Age groups

**Figure 5:**
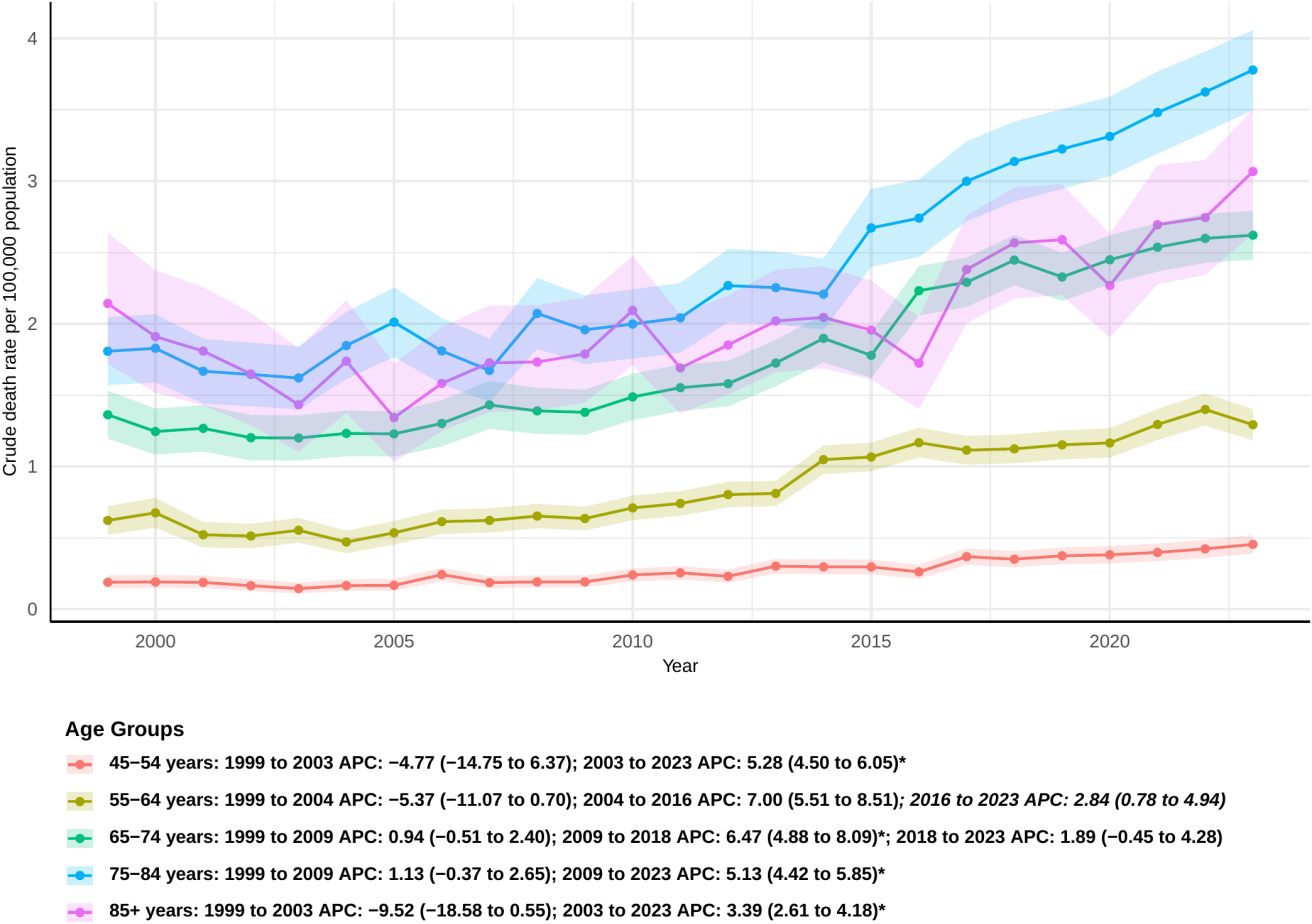
Joinpoint model of sepsis among middle-aged and elderly patients with pancreatic cancer AAMR per 100,000 residents by age groups, 1999-2023. Temporal trend segments are represented by APC values. (*APC Significant. APC, annual percentage change; AAMR, age-adjusted mortality rate).

The overall mortality rate increased across all age groups. Among individuals aged 45-54, a significant increase in mortality was observed from 2003 to 2023 (APC: 5.28; 95% CI 4.59 to 8.33). Although this age group represents the smallest cohort, it showed the largest overall increase in AAPC, with a value of 3.53 (95% CI: 1.66 to 5.44). A similar trend was noted in the 55–64 age group, where mortality rates rose steadily after 2004, leading to an AAPC of 3.10 (95% CI: 1.59 to 4.63). In the 65–74 age group, a sharp increase in mortality was observed from 2009 to 2018 [APC: 6.47 (95% CI: 4.88 to 8.09)], with an overall AAPC of 3.18 (95% CI: 2.27 to 4.10) (**Figure 5**). For individuals aged 75-84, the mortality rate significantly increased from 2009 to 2023 [APC: 5.13 (95% CI: 4.42 to 5.85)], and, from 2011 onwards, the AAMR in this group consistently remained higher than in other age groups. The 85 and older age group was the only group without a significant overall AAPC (**Supplementary Table 2**); however, a significant increase in mortality was observed from 2003 to 2023 (APC: 3.39 [95% CI: 2.61 to 4.18]).

### HHS regions

**Figure 6:**
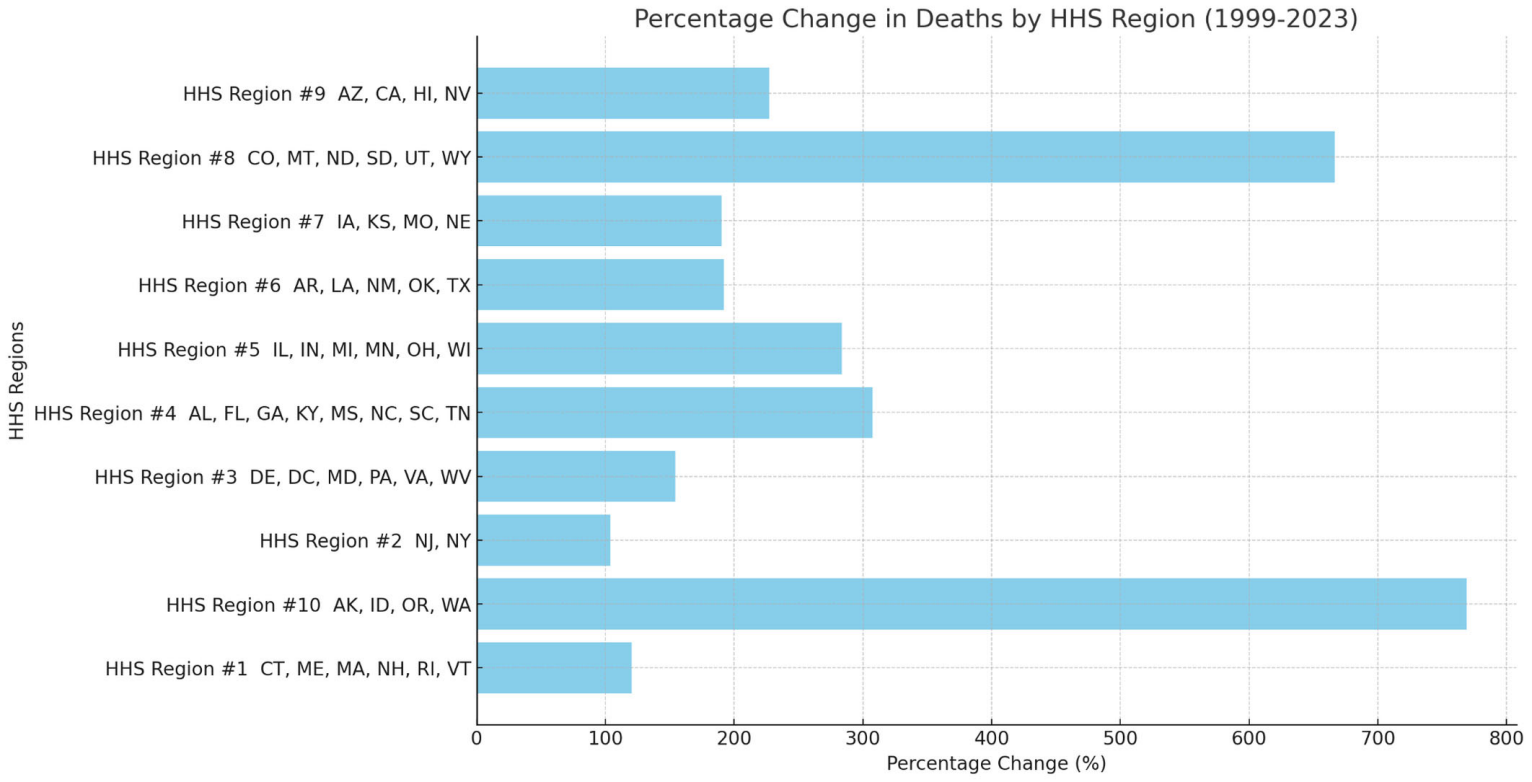
Percent change of sepsis among middle-aged and elderly patients with pancreatic cancer death by HHS regions (Bar Chart), 1999 and 2023.

**Figure 7:**
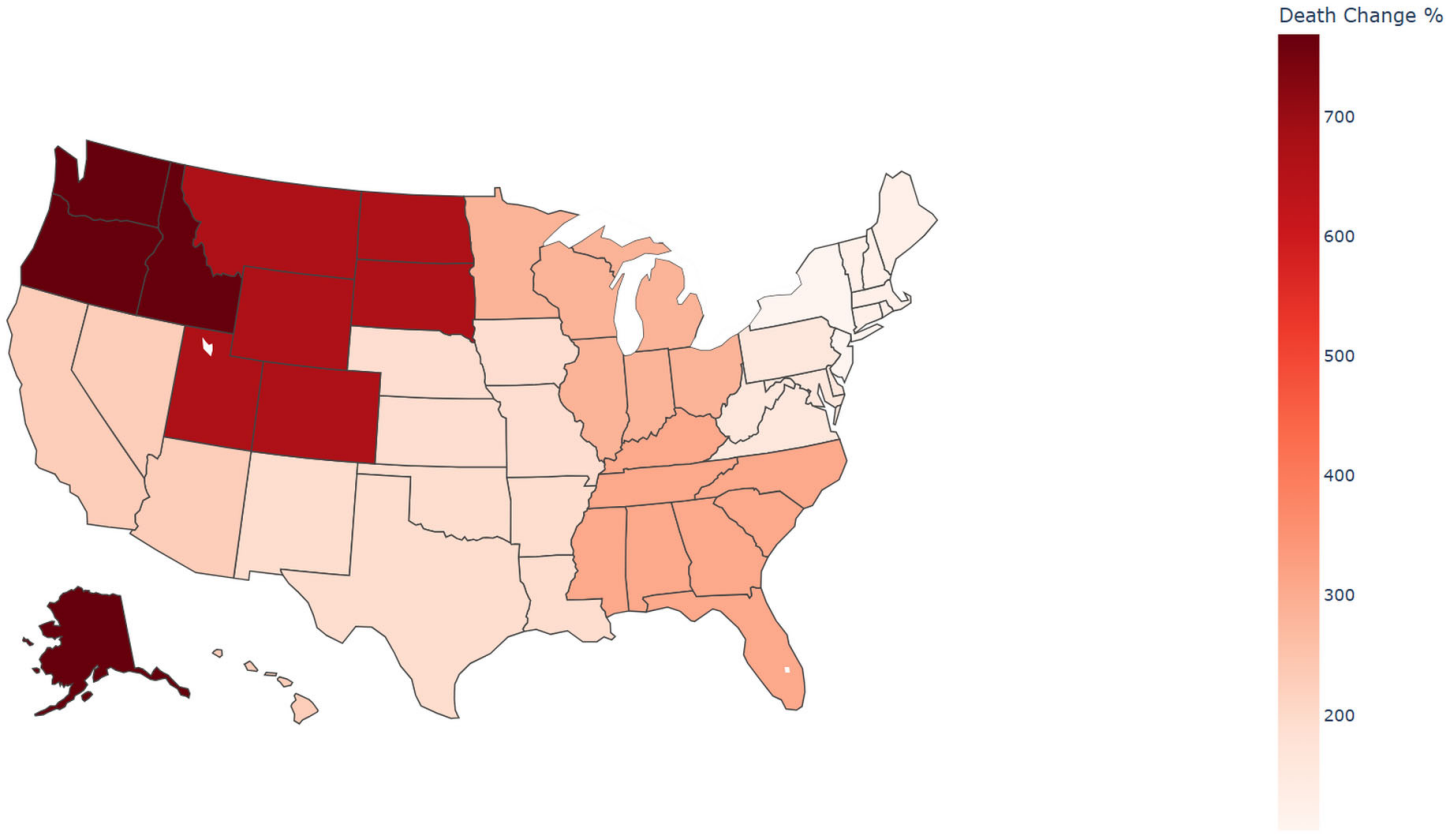
Percent change of sepsis among middle-aged and elderly patients with pancreatic cancer death by HHS regions (Choropleth Map), 1999 and 2023.

Due to sparse data or data labeled as ‘suppressed’ or ‘unreliable’ in certain states, which impacted the reliability of statistical results, the analysis was ultimately conducted by Health and Human Services (HHS) region. From 1999 to 2023, significant variations in the percentage change of sepsis-related mortality rates among pancreatic cancer patients were observed across the U.S. HHS regions(**Supplementary Table 3**). The region with the largest percentage change in mortality was HHS Region 10 (769.23%), which includes Alaska, Idaho, Oregon, and Washington. In contrast, the region with the smallest percentage change was HHS Region 2 (103.74%), encompassing New Jersey and New York(**Figures 6**, **7**). Despite these regional differences, the percentage change in mortality rates showed an upward trend across all HHS regions.

## Discussion

The research examined death rates linked to sepsis among middle-aged and older patients with pancreatic cancer from the CDC WONDER database, aiming to investigate demographic trends over time from 1999 to 2023. We defined sepsis deaths using only ICD-10 A40–A41 in order to facilitate national temporal trend comparability, as opposed to more recent coding strategies and a more expansive definition of sepsis. This is in line with the NCHS “113 Select Causes of Death” category for “Septicemia.” This might understate the number of cases, but it won’t change the trend’s trajectory.During the duration of the study, there was a steady escalation in the total AAMR, especially pronounced starting in 2003. Through joinpoint regression, three separate stages of escalation were pinpointed, highlighting the escalating incidence of sepsis among this vulnerable group. Mortality rates also increased with age, reinforcing the well-established notion that advanced age is a significant risk factor for poorer outcomes. Multiple studies have indicated that patients aged ≥65 or ≥75 years experience notably higher 90-day mortality and complication risks following pancreatic resection, with sepsis and septic shock emerging as major threats (25, 26).

When stratified by gender, significant differences were observed between male and female populations. The AAMR for males was consistently higher than for females, aligning with existing evidence that male pancreatic cancer patients face greater risks of complications and mortality. This gender disparity is especially pronounced in sepsis and its associated adverse outcomes.Additionally, biological sex itself affects immune programming: women have stronger innate and adaptive immune responses overall, partly because female immune cells are able to escape the inactivation of important innate immune genes on the X chromosome (like TLR7), which has the effect of enhancing anti-pathogen responses. Men may have less control over infection loads in middle and old age if they do not have this beneficial pathway (27, 28). Additionally, progesterone suppresses inflammatory factors prior to menopause, and estrogen (through the ERα/GPER pathway) may offer protection in middle-aged and older women by regulating the endothelial barrier, antioxidant, and anti-inflammatory signaling (29). However, E2/P4 levels quickly drop after menopause, which lessens this protective effect in women to some extent. However, men still have a higher risk of sepsis-related mortality due to their combined overall immune response and comorbidity burden, which includes obesity and type 2 diabetes (30, 31).Progesterone has been shown in animal and cell studies to downregulate oxidative stress, myeloperoxidase activity, and TNF-α/IL-6 in sepsis, indicating that it may have immunosuppressive inflammation and organ protection properties. Although these benefits decrease with postmenopausal decline in E2 levels, estrogen can also have protective effects through antioxidant defenses, macrophage regulation, and enhancements in microcirculatory perfusion and endothelial barrier function (32). The routine use of sex hormones for treating sepsis is not currently supported by these biological mechanisms, despite the fact that they offer tenable explanations for women’s lower AAMR (33).

The fatality rates associated with sepsis in pancreatic cancer patients vary markedly among different racial groups. During the study period, non-Hispanic Black or African American individuals demonstrated a significantly elevated AAMR relative to other racial groups, yielding an overall AAPC of 2.62 (95% CI: 1.76 to 3.48). This difference is due to long-term systemic racial discrimination and unfairness in healthcare, jobs, education, and the justice system, which have led to higher death rates for this group (34, 35). A comprehensive retrospective analysis utilizing the U.S. National Inpatient Sample revealed that race and gender are key determinants affecting inpatient mortality rates among individuals hospitalized for end-stage diseases, such as pancreatic cancer and sepsis. The study showed that Black patients had higher rates of death in the hospital than White patients, stayed longer in the hospital, and paid more for medical care. These differences are probably caused by a complicated mix of social, cultural, and economic variables.The study also showed that Black patients were less likely to obtain “Do Not Resuscitate” (DNR) orders. This could be because of cultural differences in how people plan for their care and make decisions regarding life-sustaining therapies when they have end-stage diseases (36). The pancreatic cancer stages of non-Hispanic Blacks and African Americans are more advanced, they receive less high-standard treatment, and they have less access to multidisciplinary care pathways and high-volume surgical centers. Following sepsis, these variables, along with a higher incidence of diabetes and obesity, increase the risk of death and disadvantage long-term survival (37).

A more in-depth look at the death rates from sepsis among pancreatic cancer patients of different races shows that Hispanic or Latino people had the highest increase in death rates. A comprehensive national investigation examining patients with febrile neutropenia (FN) and Clostridium difficile infection (CDI) yielded substantial direct evidence correlating Hispanic identity with elevated inpatient death rates. In this study, most of the patients had cancer, and the analysis found that being Hispanic was a strong predictor of inpatient death, along with other known risk factors like being older, having hematologic malignancies, and having sepsis. This study explicitly linked Hispanic identity to an increased mortality risk in cancer patients experiencing specific viral comorbidities (38).In addition to tumor-specific investigations, more general observations can also provide useful information. For example, a study on recurrent pneumococcal bacteremia discovered that all recurrent patients were either African American or Hispanic, with malignancy serving as an independent predictor of recurrence (39). Between 1999 and 2020, the mortality rates from pancreatic cancer rose more sharply among Hispanics and Latinos, indicating the combined impact of increased metabolic risks (such as obesity and type 2 diabetes), a higher incidence of late-stage medical presentations, and different treatment approaches in this demographic (40). This phenomena cannot be ascribed to a singular source; rather, it arises from the interaction of multiple aspects, including socioeconomic level, healthcare access and quality, comorbidity burden, and possibly genetic predisposition (41). The tumor and its treatment weaken the immune system, making these people more likely to get infections (42, 43). The extra risk factors in the Hispanic population also make their prognosis worse.

Among all regions, the Midwest experienced the most significant increase in deaths related to sepsis in pancreatic cancer patients. Latest studies indicate an increase in the count of acute critical diseases linked to cancer in the Midwest. Research shows a rising trend in the incidence of cardiac arrests linked to sepsis within hospitals (SA-IHCA) in this region (44). Hospitals in the Midwest, South, and West frequently encounter Acute Respiratory Distress Syndrome (ARDS), a critical and frequent sepsis complication (45). Within the expansive countryside areas of the U.S. In the Midwest, recognizing and handling sepsis presents unique challenges (46). A retrospective cohort study at a Midwest academic medical center revealed that severely sick patients with sepsis and septic shock transferred from community hospitals experienced considerable delays in receiving vital therapies compared to those directly admitted to specialist institutions (47, 48). Specifically, moved patients were markedly less likely to obtain suitable initial antibiotic treatment, and a reduced percentage got fluid resuscitation within the crucial 3-hour timeframe. These delays go against the main ideas of early goal-directed therapy for sepsis. This problem is widespread in sepsis treatment routes in the Midwest, where survivors of sepsis who have a cardiac arrest in the hospital are more likely to be moved to another hospital than to be sent home right away. Optimizing inter-hospital transfer processes and ensuring seamless continuity of care before, during, and after transfer are crucial to minimizing preventable delays and improving patient outcomes in sepsis management. The Midwest’s “metabolic geography,” which is defined by a high burden of obesity and diabetes, is associated with the greatest increase in sepsis-related mortality among patients with pancreatic cancer. Through immunological suppression, glucose excursions, and hyperglycemia brought on by stress, both can considerably worsen sepsis outcomes. The stress-induced hyperglycemia ratio (SHR) and sepsis mortality have been found to have a U/J-shaped relationship in recent studies based on intensive care unit data, indicating that abnormal glucose homeostasis is a stand-alone prognostic indicator. Patients with aberrant glucose metabolism are especially directly affected by this (49). The Midwest and South have high obesity rates, according to the CDC/NCHS report. These metabolic comorbidities, when combined with malnourishment, immunosuppression from chemoradiation, and biliary obstruction from pancreatic cancer, can readily lead to biliary/duct-related and postoperative infections, increasing the mortality rate from regional sepsis (50).

In the U.S., healthcare resources and high-risk populations are predominantly concentrated in urban areas, making sepsis a particularly prominent issue for cancer patients in these regions and posing a substantial challenge to the public health system. Studies indicate that a large number of cancer patients are treated in major urban hospitals, which also serve as critical centers for handling a variety of critically ill patients (51, 52). An analysis of the U.S. inpatient sample from 2001 to 2010 showed a 27.5% increase in admissions for emergency general surgery (EGS), with sepsis cases rising by 15%. The annual incidence rate of sepsis in this diverse patient population surpasses the total number of newly diagnosed cancer cases, with nearly 85% of patients receiving treatment in urban hospitals, underscoring the central role of urban healthcare systems in managing these cases (52).Hospital-acquired sepsis remains a significant challenge, particularly in large urban hospitals where high-risk patients are concentrated. A study conducted at four metropolitan-area hospitals found that the overall mortality rate for hospital-acquired bacteremia with urinary tract infection was as high as 30.8%, with 12.7% of deaths directly attributed to the infection (53). Almost all of these deaths occurred in high-risk patients with poor prognoses, such as those with malignancies, severe neurological disorders, or advanced liver and kidney diseases. Furthermore, another study identified specific sources of hospital-acquired infections, revealing that the insertion of central venous catheters was the strongest independent risk factor for hospital-acquired GBS infection, with an odds ratio of 30.9 (54). This risk is especially high for cancer patients who often require central venous access for chemotherapy or other treatments, highlighting the critical role of invasive medical procedures in the pathogenesis of hospital-acquired sepsis.

In contrast, the elevated death rate from sepsis in rural regions is intricately associated with the density of susceptible populations and their distinct clinical attributes. First, people with underlying comorbidities are at a higher risk in rural areas. In the same way, older people living in rural locations are more likely to die, with sepsis being a primary cause of death (55). Frailty, defined as age-related multisystem functional deterioration, is acknowledged as a critical risk factor for the onset of sepsis and septic shock in conditions such as acute pancreatitis, resulting in elevated fatality rates (56). The clinical appearance of rural patients upon admission is frequently more severe. Research within the Veterans Health Administration (VHA) framework showed a notable increase in sepsis diagnoses among patients in low-complexity rural intensive care units (ICUs) compared to those in urban ICUs, with their illness severity scores at admission being substantially higher (57). This implies that patients in rural areas may not receive intensive care until their disease has progressed, so complicating treatment and exacerbating outcomes. Numerous national studies and reviews have demonstrated that there are still differences between urban and rural areas in the United States when it comes to cancer outcomes. Changes in hospital capacity, quality implementation, and access to healthcare have been linked to slower and higher declines in cancer mortality in rural areas (58, 59).

In the future, dealing with sepsis-related deaths in people with pancreatic cancer will need a wide-ranging, multi-faceted approach. First, build a precise stratification model, comprehensively considering systemic factors, biological characteristics, comorbidities, and treatment exposure, as well as preempt prevention and monitoring; Second, surgical methods should always be improved, with a focus on minimally invasive procedures and other methods that lower the risk of infection while still being effective for cancer treatment. Third, infection control throughout the preoperative period and chemotherapy needs to be improved, with consistent procedures for prevention, early detection, and quick action. The ultimate objective is to diminish the incidence of sepsis through multidisciplinary collaboration, so enabling a larger cohort of pancreatic cancer patients to effectively finish their treatment regimens and optimize survival results.

Our study has a number of drawbacks. First, using ICD-10 death certificate codes exclusively could result in underreporting or incorrect classification, which would introduce measurement bias and compromise the precision of trend estimates. Second, there is a lack of individual-level clinical information in the data, including comorbidity severity, unique treatment exposures (such as chemotherapy, stenting, surgery, and intensive care unit admission), and cancer stage. This makes it difficult to control for potential confounders and may result in residual confounding. Additionally, the study was limited in its ability to draw conclusions regarding causal mechanisms due to the death certificate data’s lack of temporal ordering and fine temporal resolution, which made it impossible to discern sequential relationships between sepsis and cancer-related complications or treatments. Finally, the Joinpoint regression procedure’s crude mortality rates were the only data available for age group analyses. Inadequate information from the CDC WONDER database limited state analyses.

## Conclusion

In summary, our research reveals a notable and continuous rise in death rates due to sepsis among middle-aged and older patients with pancreatic cancer in the U.S. between 1999 and 2023. Mortality’s impact is unevenly spread, showing notably elevated rates in Black or African American patients, men, those in the Midwest and cities, and the aged. The results highlight not just the biological severity of pancreatic cancer and its unfavorable outlook linked to sepsis, but also emphasize the inadequacies in existing preventive approaches, healthcare service inequalities, and management deficiencies.

Considering the persistently elevated risk of sepsis in this group, the findings support the creation of tailored sepsis risk models for pancreatic cancer in middle-aged and older patients. Furthermore, there is a demand for the enhanced incorporation of practical data into preventive measures and specific programs designed to diminish disparities in diagnosis and care. These efforts are expected to improve the challenging situation of pancreatic cancer complicated by sepsis, leading to longer survival and an enhanced quality of life for patients, while also helping to reverse the rising trend of sepsis-related deaths.

## Data Availability

The original contributions presented in the study are included in the article/**Supplementary Material**. Further inquiries can be directed to the corresponding author.
